# Draft Genome Sequence of *Arenibacter* sp. Strain C-21, an Iodine-Accumulating Bacterium Isolated from Surface Marine Sediment

**DOI:** 10.1128/genomeA.01155-16

**Published:** 2016-10-13

**Authors:** Kohei Ito, Nobuyoshi Nakajima, Shigeki Yamamura, Masaru Tomita, Haruo Suzuki, Seigo Amachi

**Affiliations:** a Institute for Advanced Biosciences, Keio University, Fujisawa, Kanagawa, Japan; b Center for Environmental Biology and Ecosystem Studies, National Institute for Environmental Studies, Tsukuba, Ibaraki, Japan; c Center for Regional Environmental Research, National Institute for Environmental Studies, Tsukuba, Ibaraki, Japan; d Graduate School of Horticulture, Chiba University, Matsudo City, Chiba, Japan

## Abstract

*Arenibacter* sp. strain C-21, isolated from surface marine sediment of Japan, accumulates iodine in the presence of glucose and iodide (I^-^). We report here the draft genome sequence of this strain to provide insight into the molecular mechanism underlying its iodine-accumulating ability.

## GENOME ANNOUNCEMENT

Bacteria may play significant roles in the biogeochemical cycling of iodine through volatilization, oxidation, reduction, and accumulation of this element (1). Previously, we isolated *Arenibacter* sp. strain C-21 from surface marine sediment collected from Sagami Bay, Kanagawa, Japan (2). When strain C-21 was grown in the presence of 0.1 µM iodide ion (I^-^), it accumulated iodine with a maximum concentration factor of 5.5 × 10^3^. Subsequent studies revealed that glucose, oxygen, and calcium ion were required for iodine uptake by this strain (3). However, glucose-independent uptake of iodine was observed if a hydrogen peroxide-generating system or iodide-oxidizing system was present. A possible mechanism of iodine uptake and accumulation by strain C-21 was proposed in which a membrane-bound glucose oxidase and haloperoxidase are involved (3).

*Arenibacter* sp. strain C-21 was grown in Marine broth 2216 (Difco), and DNA was extracted using a DNeasy blood and tissue kit (Qiagen, Hilden, Germany). Whole-genome sequencing was performed using paired-end sequencing on an Illumina MiSeq. The sequencer produced 300-bp paired-end reads that were obtained from 550-bp inserts. The quality of the reads was checked using PRINSEQ (4). *De novo* genome assembly was performed using Velvet 1.2.10 (5, 6). The resulting assembly contains 92 contigs consisting of 5,667,524 bp, with a G+C content of 39.7%. Genome annotation was performed using Prokka version 1.11, which is a pipeline comprising several bioinformatic tools (7). Briefly, Aragorn (8) detected 39 tRNA genes, Barrnap predicted four (one of 16S, one of 23S, and two of 5S) rRNA genes, and Prodigal (9) identified 4,766 protein-coding DNA sequences (CDSs), of which 438 contained signal peptides identified using SignalP (10). Of the 4,766 proteins, 1,578 were hypothetical proteins of unknown function, 2,140 proteins were annotated by UniProtKB (11), 761 proteins were annotated by Pfam (12), 343 proteins were annotated by NCBI’s Conserved Domain Database (CDD) (13), and 57 proteins were annotated by HAMAP (14). For example, of the annotated proteins, 39 were SusD family proteins (Pfam: PF07980), 35 were arylsulfatase (UniProtKB: P51691 and Q0TUK6), and 18 were vitamin B_12_ transporter BtuB (HAMAP: MF_01531). At least 28 genes coding for *c*-type cytochromes were identified in the genome. A BLASTp search (15, 16) revealed that the genome contained at least five genes annotated as PAP2 superfamily proteins (Pfam: PF01569) that are homologous to vanadium iodoperoxidase from *Zobellia galactanivorans* (PDB: 4USZ_A) (17). The draft genome sequence of *Arenibacter* sp. strain C-21 represents a valuable resource for future comparative functional genomic studies.

### Accession number(s).

*Arenibacter* sp. strain C-21 whole-genome shotgun project has been deposited at DDBJ under the accession number BDGL00000000. The version described in this paper is the first version, BDGL01000000, which consists of sequences BDGL01000001 to BDGL01000092.
